# Neoehrlichiosis in Symptomatic Immunocompetent Child, South Africa

**DOI:** 10.3201/eid2902.221451

**Published:** 2023-02

**Authors:** Colleen Bamford, Lucille H. Blumberg, Michelle Bosman, John Frean, Kim G.P. Hoek, Janet Miles, Charlotte Sriruttan, Ilse Vorster, Marinda C. Oosthuizen

**Affiliations:** Pathcare, East London, South Africa (C. Bamford); University of Cape Town, Cape Town, South Africa (C. Bamford);; National Institute for Communicable Diseases, Johannesburg, South Africa (L.H. Blumberg, J. Frean, C. Sriruttan);; University of Pretoria, Onderstepoort, South Africa (L.H. Blumberg, J. Frean, I. Vorster, M.C. Oosthuizen);; University of Stellenbosch, Tygerberg, South Africa (L.H. Blumberg, K.G.P. Hoek);; Ampath, East London, South Africa (M. Bosman);; University of the Witwatersrand, Johannesburg, South Africa (J. Frean);; PathCare Reference Laboratory, Cape Town (K.G.P. Hoek);; East London, South Africa (J. Miles)

**Keywords:** neoehrlichiosis, zoonoses, vector-borne infections, bacteria, Candidatus Neoehrlichia, ehrlichiosis, tickborne infections, South Africa

## Abstract

We describe a case of neoehrlichiosis in an immunocompetent child with acute febrile illness in South Africa. Neoehrlichiosis was diagnosed by PCR on 16S rDNA from bone marrow aspirate. Phylogenetic analysis indicated an organism closely related to *Candidatus* Neoehrlichia. Clinicians should be aware of possible ehrlichiosis even in immunocompetent patients.

*Ehrlichia* species and related organisms are obligate intracellular membrane-bound bacteria transmitted via ticks between a range of mammalian animal hosts, including humans. In recent years, taxonomy and phylogeny of these bacterial species has been revised. The family Anaplasmataceae comprises 5 genera: *Ehrlichia*, *Anaplasma*, *Neorickettsia*, *Neoehrlichia*, and *Wolbachia*. However, the number of recognized species and candidate species has substantially increased in recent years. We describe a case of neoehrlichiosis in an immunocompetent child in South Africa. 

## The Study

A previously healthy 9-year-old boy from Eastern Cape Province, South Africa, was admitted to the hospital with a 3-week history of fever, back pain, and myalgia. Symptoms failed to respond to a course of azithromycin prescribed by his general practitioner for suspected tickbite fever. The child lived on a farm and was exposed to a variety of domestic companion and farm animals, but he had no history of recent tick bites or travel.

At admission, results of a physical examination were unremarkable, except for a temperature of >40°C. The patient had no rash, skin lesions, lymphadenopathy, or clinically detectable hepatosplenomegaly. Laboratory investigation results were unremarkable (Table). The patient received empiric treatment with ceftriaxone for 5 days, but symptoms did not improve.

**Table:** Laboratory and imaging investigations in a case of neoehrlichiosis in a symptomatic immunocompetent child, South Africa

Investigation	Patient result	Reference value or range
C-reactive protein, mg/L	17.2	<5
Leukocytes, × 10^9^ cells/L	15.8	4.5–13.5
Neutrophils, × 10^9^ cells/L	10.98	1.50–8.5
Lymphocytes, × 10^9^ cells/L	2.99	1.0–6.5
Monocytes, × 10^9^ cells/L	1.50	0–0.8
Platelets, × 10^9^ cells /L	219	140–420
Hemoglobin, g/dL	12.7	11.5–15.5
Blood cultures	No growth	NA
Urine culture	No growth	NA
*Rickettsia conorii/R. africae* serology and PCR on blood	Negative	NA
SARS-CoV-2 PCR, nasopharyngeal swab and serology	Negative	NA
Cytomegalovirus PCR and serology	Negative	NA
Epstein-Barr serology	Negative	NA
Widal test	Negative	NA
Mastazyme *Brucella* ELISA	IgM and IgG equivocal	NA
Multiplex PCR for atypical and viral respiratory pathogens*	Negative	NA
QuantiFERON Gold test for tuberculosis†	Negative	NA
Imaging		
Ultrasound of the abdomen	15 cm spleen	10–11 cm at this age
Chest radiograph	No abnormalities detected	NA
Cervical and thoracic spine radiograph	No abnormalities detected	NA
Transthoracic echocardiography	No abnormalities detected	NA
Magnetic resonance imaging spine	No abnormalities detected	NA

*FilmArray RP2.1plus (BioFire Diagnostics, https://www.biofiredx.com). †QuantiFERON, https://www.quantiferon.com.

Clinicians considered brucellosis because of initial equivocal *Brucella* serologic test results, despite absence of definite exposure history, and began treatment with doxycycline, rifampin, and gentamicin. The patient gradually experienced partial improvement over the next week.

Clinicians performed bone marrow aspirate and trephine to exclude malignancy and investigate possible brucellosis; results showed a reactive inflammatory background. PCR for *Brucella* spp. was negative, and cultures yielded no growth. However, PCR and sequencing of a 598-bp section of the hypervariable 5 to 8 regions of the 16S rRNA gene (*1*), revealed probable *Ehrlichia* species.

Clinicians discontinued rifampin and gentamicin but continued doxycycline for a total of 14 days, and the symptoms completely resolved. The child remained well at follow-up 12 months later. The child’s mother gave written informed consent for publication, and approval was granted by the Human Research Ethics Committee of the University of Cape Town (reference no. 551/2022).

We subjected the bone marrow samples to a reverse line blot hybridization assay for simultaneous detection and differentiation of the most common *Ehrlichia* and *Anaplasma* spp. by using a PCR targeting the 16S rRNA gene (*2*,*3*). PCR product hybridized with the *Ehrlichia/Anaplasma* genus-specific probe but not with any species-specific probes, suggesting a novel species or variant of a known species.

We PCR amplified the near full-length 16S rRNA gene (1,467 bp) using primers fD1 and rP2 (*4*), cloned and sequenced the resultant amplicons. We assembled and edited the generated sequences using the Staden Package (https://staden.sourceforge.net). The consensus sequence was deposited in Genbank (accession no. OP208838). BLASTn (https://blast.ncbi.nlm.nih.gov) homology search results revealed no identical sequences in the public databases. The most closely related sequences, at 96%–98% identity, were various *Candidatus* Neoehrlichia 16S rDNA sequences, including *Candidatus* N. mikurensis (strain TK4456^R^), *Candidatus* N. lotoris (strain RAC413^R^), and *Candidatus* N. arcana (strain HT94^R^) (Figure).

**Figure:**
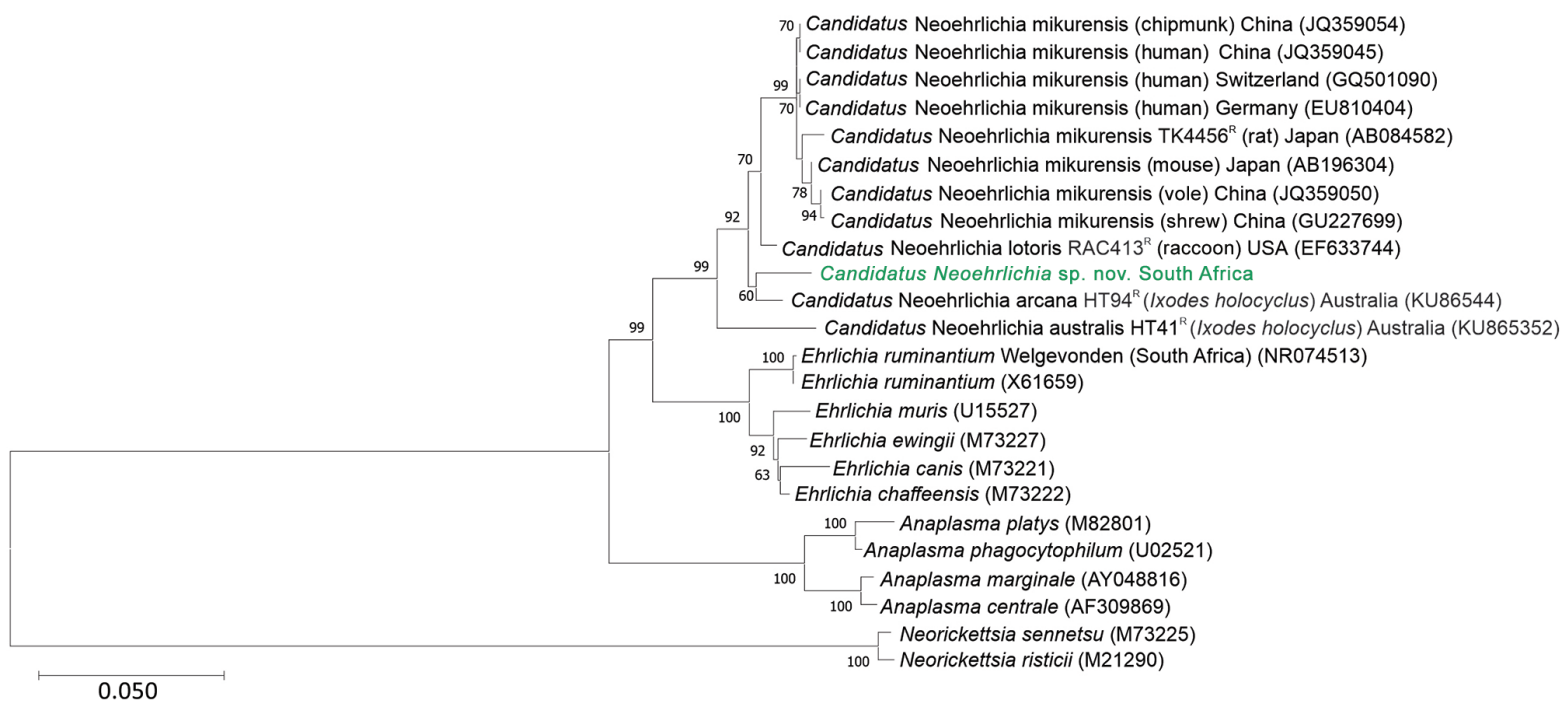
Phylogenetic tree of *Candidatus* Neoehrlichia species detected in a case of neoehrlichiosis in a symptomatic immunocompetent child, South Africa. We used Kimura 2-parameter plus gamma plus invariable site substitution model in MEGA X (https://www.megasoftware.net) to infer a maximum-likelihood phylogenetic tree in combination with the bootstrap method using 1,000 replicates. Green bold text indicates *Candidatus* Neoehrlichia species detected from the patient in this case-study; we compared this isolate to other Anaplasmataceae species detected from ticks, humans, and mammals available in GenBank (indicated by species name and GenBank accession number; host species and country are given for other *Candidatus* Neoehrlichia species). Scale bar indicates nucleotide substitutions per site.

We aligned the consensus sequence with closely related 16S rDNA sequences from Genbank by using ClustalX (http://www.clustal.org). We manually examined the alignments and truncated to smallest sequence size, a 1,264-bp *Candidatus* N. australis sequence, by using BioEdit 7.2.5 (https://bioedit.software.informer.com). We used MEGA X (https://www.megasoftware.net) to determine Kimura 2-parameter plus gamma plus invariable site substitution as the best-fit model. We used it in combination with the bootstrap method using 1,000 replicates to infer a maximum-likelihood phylogenetic tree, which indicated that our obtained sequence clustered with *Candidatus* N. arcana, although with low bootstrap support (60%) (Figure). The sequence was closely related to, but distinct from, *Candidatus* N. lotoris and *Candidatus* N. mikurensis. As previously shown (*5*,*6*), *Candidatus* Neoehrlichia mikurensis, *Candidatus* N. lotoris, and *Candidatus* N. arcana are distinct from other genera in the Anaplasmataceae family and form a well-supported sister clade to the genus *Ehrlichia*. On the basis of whole-genome sequencing, *Candidatus* N. mikurensis appears to be more closely related to *Ehrlichia chaffeensis* than to *E. ruminantium*, suggesting that establishing the genus *Neoehrlichia* might have been premature (*7*).

We calculated estimated evolutionary divergence by determining the number of nucleotide differences between similar sequences. We removed all ambiguous positions for each sequence pair (pairwise deletion option), which left a total of 1,264 positions in the final dataset. Our obtained sequence differed from GenBank reference strains by 23 bp for *Candidatus* N. lotoris, 24 bp for *Candidatus* N. arcana, and 34 bp for *Candidatus* N. mikurensis. These reference species differ by 46–50 bp from each other. Our findings confirmed that the ehrlichial organism we identified is a member of the family Anaplasmataceae and most closely related to, but distinct from, *Candidatus* N. lotoris and *Candidatus* N. arcana. Until appropriate type-material can be deposited and the species is formally described, we will refer to this novel organism as *Candidatus* Neoehrlichia sp. South Africa (OP208838).

## Conclusions

We report a novel ehrlichial organism, most closely related to the genus *Candidatus* Neoehrlichia, a sister genus to *Anaplasma* and *Ehrlichia*, detected in the bone marrow aspirate of an acutely febrile immunocompetent child from South Africa. *Candidatus* N. lotoris has been described from raccoons in Georgia, USA, and an associated tick species (*7*), but it has not been detected in humans or any other wild vertebrate species. *Candidatus* N. arcana has been described from *Ixodes holocyclus* ticks in Australia (*8*), but its pathogenic significance has not yet been determined (*6*). *Candidatus* N. mikurensis was described in 2004 (*5*), and a human case was reported in 2010 (*9*). *Candidatus* N. mikurensis appears to be widely distributed in Europe (*10*), especially in central Europe and Scandinavia, as well as in Asia (*11*). Data from Africa are limited, but *Candidatus* N. mikurensis has been isolated from dog ticks in Nigeria (*12*). The host reservoir of *Candidatus* N. mikurensis is small mammals, and its vectors include *Ixodes* and *Haemaphysalis* tick species (*10*).

Most human neoehrlichia infections described to date have been caused by *Candidatus* N. mikurensis, and clinical findings are usually nonspecific, including fever, headache, and myalgia; however, vasculitis and thrombo-embolic events can occur (*13*). Infections are more commonly recognized in immunocompromised patients; infections in immunocompetent patients can be mild or asymptomatic (*14*). 

Another probable clinical neoehrlichia case from Africa was reported in 2013 in a traveler who returned to Denmark from Tanzania (*15*). The researchers described a potentially novel neoehrlichia species isolated from the patient’s blood. On the basis of analysis of short, 300–345-bp fragments, the species was closely related to both *Candidatus* N. lotoris and *Candidatus* N. mikurensis (*15*).

Ehrlichiae and related organisms are usually detected via molecular methods, including PCR of 16S rRNA, or *groEL/groESL* and *gltA* genes. Application of new molecular methods is leading to an explosion in discovery of related novel species or variants, as noted in Australia (*6*) and elsewhere. Recognition of associated human disease is increasing, especially in immunocompetent patients. Further elucidation of the taxonomic status of the novel ehrlichial organism identified in this case study would require whole-genome sequencing or multilocus sequence analysis targeting genes for which *Neoehrlichia* gene sequences are available, including 16S rRNA or *ftsZ*, *gatB*, *groEL*, and *lipA*.

Although tickborne rickettsial infections are common in South Africa, Anaplasmataceae infections likely are undiagnosed because of lack of awareness among clinicians and unavailability of laboratory tests. Of note, *Ehrlichia* spp. are not susceptible to macrolides and our case would not have been recognized if the patient had initially been treated with doxycycline. 

In conclusion, our case represents an expansion of the known range of neoehrlichiosis. Clinicians should be aware of neoehrlichia as a possible tickborne cause of acute febrile illness in Africa.
